# Family screening for a novel ATP7B gene mutation, c.2335T>G, in the South of Iran

**Published:** 2014-03-15

**Authors:** J Manoochehri, R Masoumi Dehshiri, H Faraji, S Mohammadi, H Dastsooz, T Moradi, E Rezaei, Kh Sadeghi, M Fardaei

**Affiliations:** 1 Department of Medical Genetics, Medical School, Shiraz University of Medical Sciences, Shiraz, Iran; 2 Comprehensive Medical Genetics Centre, Shiraz, Iran; 3 Health Policy Research Center, Shahid Sadoughi University of Medical Sciences and Health Services, Yazd, Iran.; 4 Department of Molecular Medicine, School of Advanced Medical Sciences and Technologies, Shiraz University of Medical Sciences, Shiraz, Iran; 5 Student Research Committee, Shiraz University of Medical Sciences, Shiraz, Iran

**Keywords:** ARMS-PCR, c.2335T>G, p.Trp779Gly, South of Iran, Wilson disease

## Abstract

**Background:**

Wilson disease (WD) is a rare autosomal recessive disorder, which leads to copper metabolism, due to mutations in ATP7B gene. The gene responsible for WD consists of 21 exons that span a genomic region of about 80 kb and encodes a copper transporting P-type ATPase (ATP7B), a protein consisting of 1465 amino acids. Identifying mutation in ATP7B gene is important to find carrier individuals for proper counseling. A novel mutation in exon 8 of ATP7B gene, c.2335T>G (p.Trp779Gly), with severe neuropsychiatric condition in the South of Iran, was recently identified.

The aim of this study was to screen 120 individuals from a large family using a simple amplification refractory mutation system PCR (ARMS-PCR) for carrier screening in the South of Iran.

**Materials and Methods:**

120 individuals from family relatives of an index case in the Nasr Abad, south of Iran, were studied for screening of the c.2335T>G mutation. One patient with homozygous mutation and one homozygous normal individual were used as controls in this experiment.

**Results:**

Altogether, 16 out of 120 (13.3%) individuals within this region had heterozygous mutation. One individual with homozygote mutation was also identified.

**Conclusion:**

Identification of carriers in families with affected individuals is of great importance for counseling before marriage. The results of this study can be used for further counseling programs in this population.

## Introduction

Wilson disease (WD) is a rare autosomal recessive disorder of copper metabolism (1). Its prevalence has not been studied in Iranian population. Patients with WD suffer from acute liver failure, hemolytic anemia, chronic liver diseases, neurological and renal diseases. Kayser-Fleischer rings were also reported in WD patients (1-4). WD is caused by mutation at ATP7B gene which encodes a transmembrane protein, a copper transporting P-type ATPase containing 1465amino acids, which involves in biliary copper excretion and incorporation of copper into ceruloplasmin (5-8). Due to impaired ATP7B protein, accumulation of copper occurs in different tissues, resulting in clinical presentation of WD (8-9). 

WD gene, which is located on13q14, consists of 21 exons. More than 500 mutations for the ATP7B gene are listed in the Wilson Disease mutation database (database maintained by the University of Alberta: www.wilsondisease.med.ualberta.ca/search3.asp). The majority of ATP7B mutations are confined to a single family, which only a few have been described as a common mutation in a few population (10-15). Exons 8, 14, 18, 2 could be the mutation hotspots of ATP7B gene, so their mutation screening is very necessary.

 In previous study, we identified a new ATP7B gene mutation, c.2335T>G (p.Trp779Gly), in exon 8, which was associated with severe neurological manifestations in patients with this mutation (4). Once diagnosis of WD is made in an index patient, evaluation of his/her family and relatives is mandatory. An ARMS PCR can be used as a suitable technique to detect this mutation in this region. Direct mutation detection by using this approach is rapid and very helpful, if the mutation occurs with a reasonable frequency in the population. 

Currently, mutation analysis is the only reliable approach to screen the family and relatives of an index case with known mutations. Identifying mutation in ATP7B gene in this region is important to find carrier individuals for proper counseling. The aim of this study was to establish a screening for c.2335T>G mutation on the basis of family studies of relatives of an index case in the south of Iran.

## Materials and Methods

120 individuals from family relatives of an index case in Nasr Abad, south of Iran, where the new mutation was first identified, were studied for screening of the c.2335T>G mutation. One normal homozygote control (without mutation) and the patient (as a positive control, with homozygote mutation) were also enrolled in this study. Individuals involved in this study, signed a consent form approved by the institutional ethical committee in Shiraz University of medical sciences.

Genomic DNA was isolated from the peripheral blood lymphocytes by AccuPrep Genomic DNA Extraction Kit (Cinnapure DNA kit) according to manufacturer's recommendations. DNA concentration was measured by NanoDrop (ND1000, USA) and the extracted DNA was stored at -20ºC until use. A simple amplification refractory mutation system PCR (ARMS-PCR) was established to study this mutation. To discriminate het¬ero- and homozygote variants by ARMS-PCR, three primers specific for exons 8 were designed, where the nucleotide changes for the mutation and normal variant was localized. The PCR products obtained from these primers was 294 bp in size (Table 1). The primer specific for nor¬mal DNA was different from the mutated DNA at their 3′ end nucleotides. In this ARMS-PCR system, a heterozygote mutation would be amplified with both normal and mutant primers, but a homozygote mutation would be only ampli¬fied with the primer specific for the mutation. This primer pair was designed, and evaluated on the basis of the ATP7B genomic sequence (GenBank accession no: NG_008806) using NCBI-BLAST and PrimerPlex 2.50 (Table 1).

By the introduction of an addi¬tional mismatch at the third nucleotide from the 3' end of the primer, the specificity of the ARMS PCR primers was enhanced. DNA sample with c.2335T> G was used to evaluate the efficiency of ARMS–PCR Then the method was used for genotyping of the 120 individuals.

PCR ampliﬁcations were carried out using ABI thermocycler. Reaction conditions were 3 µl DNA (200 ng), 1 µl of each primer (20pmol), 1µl dNTP (0.1 mM), 0.3 µl Taq polymerase, 5 µl 10x ViBuffer A, 1.75 µl MgCl2, 36.95 µl H2O in a total volume of 50 ml. 

All ampliﬁcations consisted of 95°C for 5 min, then 35 cycles of denaturing at 95 ° C for 1 min, annealing at 66°C for 30s, and extension at 72°C for 30s followed by a 5 min extension at 72°C. Following amplification, 10 µl of the PCR reaction was electrophoresed along with a 100-bp ladder on a 2% agarose gel and visualized under the Ultra Violet transillumination.

According to this issue that serum ceruloplasmin can be considered as diagnostic marker for Wilson disease, its concentration was also measured in carrier cases and some normal individuals. 

To confirm ARMS PCR results, heterozygote and homozygous samples identified by this method were also subjected to direct DNA sequencing. 

## Results

**Figure 1:**
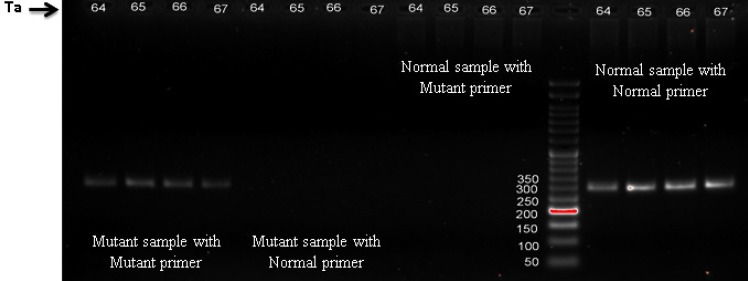
Gel electrophoresis of ARMS PCR in temperature gradient method for normal and mutant primers. Line 1-4: PCR of mutant sample with mutant primer. Line 5-8: PCR of mutant sample with normal primer. Line 9-12: PCR of normal sample with mutant primer. Line 14-17: PCR of normal sample with normal primer. 50bp DNA ladder is depicted in thirteenth line. Four Annealing temperatures (Ta) in this method were 64, 65, 66, and 67°C which depicted above each line

**Figure 2:**
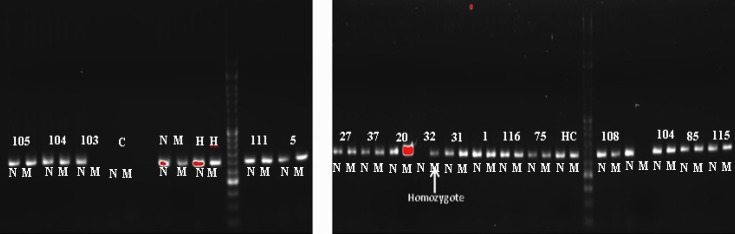
Gel electrophoresis of ARMS PCR for exon 8 of ATP7B gene at Ta of 66°C. Heterozygote samples show normal and mutant PCR bands. Sample with homozygote mutation shows only mutant PCR band (sample 32). N: PCR with normal primer, M: PCR with mutant primer. 50bp DNA ladder is depicted in middle line. H: Heterozygote control, C: Control

**Figure 3:**
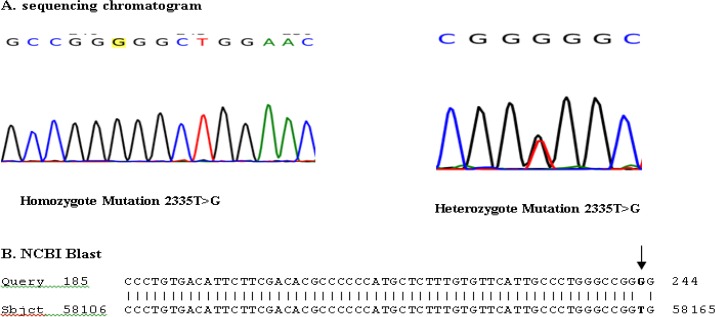
Sequencing results from homozygote and heterozygote cases. A: Sequencing chromatogram from heterozygote and homozygote cases. B: NCBI Blast of patient sequence with ATP7B reference gene show homozygote 2335T>G mutation. Query: Patient sequence, Sbjct: ATP7B gene

**Figure 4:**
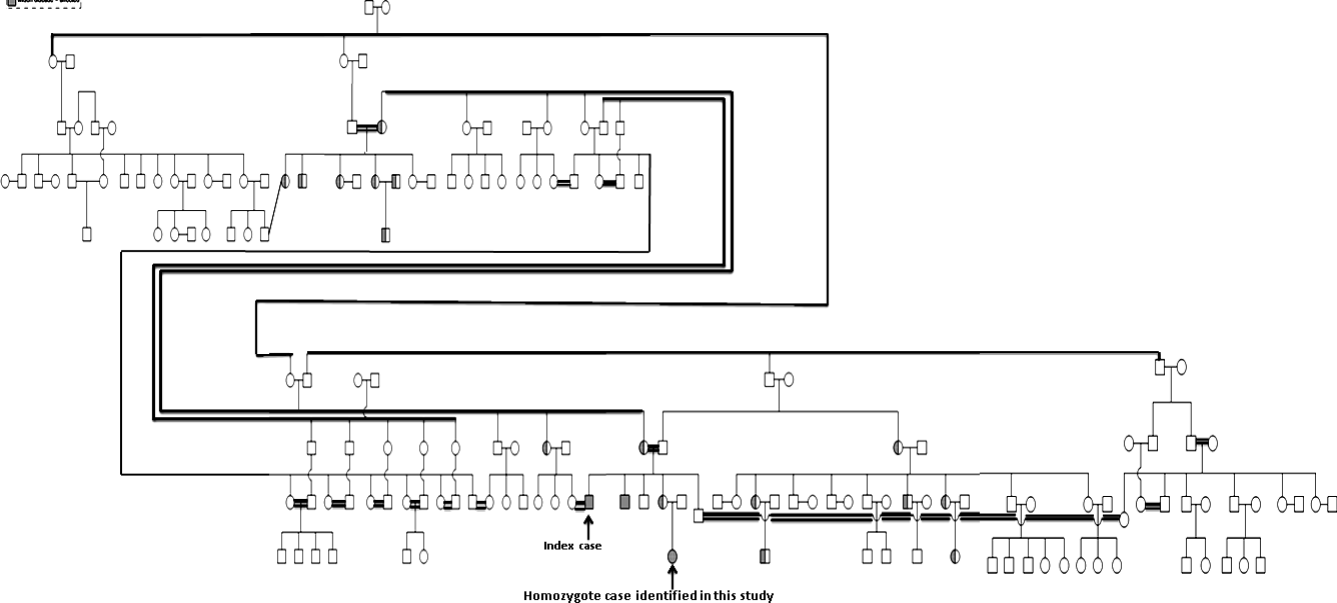
A very large Pedigree of the consanguineous family with affected WD patients from the South of Iran. Affected males and females are indicated with filled squares and circles, respectively; Heterozygote individuals are indicated with a half-filled circle or half-filled square; Double connecting lines indicate consanguineous marriages

120 individuals were screened for c.2335T > G mutation of ATP7B gene by ARMS PCR method. DNA from sample without mutation as a normal homozygote and the patient as mutant homozygote control was used as controls. The ARMS was then standardized on these DNA controls. Appropriate PCR annealing temperatures for normal and mutant primers were achieved using a temperature gradient method on the homozygote normal and mutant DNA in four PCR reactions. In this way, homozygote mutant sample shows band with mutant primer but no band with normal primer and normal samples show band with normal primer but no bands with mutant primer (Fig1). The method was then tested on 120 individuals using mutant and normal primers in separate reactions in annealing temperature of 66°C. ARMS-PCR revealed that there were 16 out of 120 (13.3%) individuals with heterozygous c.2335T>G mutation and one case with the homozygote mutation. As shown in Figure 2, cases with the heterozygous c.2335T>G mutation show positive bands for both normal and mutant primers. But the case with the homozygous mutation showed the related mutant band, and normal individuals showed only normal band (Fig 2). All ARMS PCR results are given in Table 2. Presence of this heterozygous and homozygous mutation was confirmed by direct DNA sequencing (Fig 3). Laboratory findings showed that heterozygote carriers and homozygote patient had a decreased serum ceruloplasmin concentration (Table 3). As shown in Fig 4, pedigree of this large consanguineous family with WD in the South of Iran traced back to a single pair of ancestors. All heterozygote carriers and affected cases are shown in this pedigree. 

**Table I:** ARMS PCR Primer used in this study

**EXON**	**Sequence (5'>3')**	**Product Size (bp)**	**Annealing Temperature**
**8F**	TCGCTCATTGAACTCTCCTCCCT	**294**	**66** **°C**
**8Rn**	ACCTTTGCCAAGTGTTCCAGTCA		
**8Rm**	ACCTTTGCCAAGTGTTCCAGAC***C***		

Rn: Normal Reverse Primer, Rm: Mutant Reverse Primer. Underlined letter indicates normal nucleotide. Mutant nucleotide is in italic and bold letter.

**Table II:** Samples with heterozygote and homozygote c.2335T>G mutation detected by ARMS PCR

**ARMS PCR with mutant primer**	**ARMS PCR with normal primer**	**Total cases**
**+**	**+**	**16 **
**+**	**-**	**1 **

**Table III:** Serum Ceruloplasmin levels in some normal individuals and all heterozygote carriers

**Serum Ceruloplasmin** **g/L** **)** **)**	**Genotype**	**Case No.**
**0.219**	Normal	**46**
**0.218**	Normal	**48**
**0.224**	Normal	**50**
**0.261**	Normal	**59**
**0.738**	Normal	**87**
**0.273**	Normal	**96**
**0.144**	Carrier	**1**
**0.151**	Carrier	**5**
**0.157**	Carrier	**20**
**0.195**	Carrier	**27**
**0.142**	Carrier	**31**
**0.139**	Carrier	**37**
**0.157**	Carrier	**103**
**0.141**	Carrier	**104**
**0.143**	Carrier	**105**
**0.176**	Carrier	**108**
**0.024**	**Homozygous**	**32**

## Discussion

In our previous study, a new ATP7B gene mutation, c.2335T>G, was identified in exon 8 (4). Current study is the first family investigation of a new mutation frequency using ARMS-PCR in Nasr Abad, a village in Fars province, south of Iran. Carrier frequency of the mutation for this population was 13.33%. Upon comparison of our results with other countries, the rare mutations of the ATP7B gene such as c.2335T>G occurred more in our population. 

According to our data, c.2335T>G mutation may be associated with decreased ceruloplasmin level in heterozygote carrier and can be used as a screening test for Wilson disease in this region. However, to confirm this claim, it is needed to screen more heterozygote carrier for this biochemical test.

Mutation screening approaches such as ARMS PCR have been used to study populations with high frequencies of the most common mutations of certain diseases (16-17). So, to investigate the common mutation in this small region, the ARMS PCR can be very useful and cost-effective as an initial genetic testing. To obtain the best results of ARMS assay, design of primer was very appropriate so that the melting temperature of primers was very similar, and also all primer pairs had similar concentrations in the final reaction. A temperature gradient method wasalso used to eliminate false positive results. 

The main reason for homozygosity in patients with recessive disorders is mutation inherited from a common ancestor resulted from consanguineous marriage. As can be seen in the results (Fig 4), having an affected child is due to family relative to a single pair of ancestors carrying a mutation. If an individual carries a mutation, the probability that third-degree relatives, such as cousins, share same allele is 1/8. So identification of carriers in families with affected individuals is of great importance for counseling before marriage. The results of this study can be used for prognosis and further counseling programs in this population. In such population, genetic diagnosis and counseling according to their carrier status should be proposed to all individual at risk for WD, to allow earlier treatment of affected cases.

## Conclusion

Overall, in this study, the ARMS PCR definitely detected cases with homozygous and heterozygous mutation. The high accuracy of this ARMS assay was confirmed with exact identification of this mutation in the samples. 
